# Gastric Glomus Tumor Diagnosed by Upper Endoscopy

**DOI:** 10.7759/cureus.20703

**Published:** 2021-12-26

**Authors:** Tim Brotherton, Gebran Khneizer, Eugene Nwankwo, Irfan Yasin, Mike Giacaman

**Affiliations:** 1 Internal Medicine, Saint Louis University Hospital, St. Louis, USA; 2 Gastroenterology and Hepatology, Saint Louis University Hospital, St. Louis, USA; 3 Pathology, Saint Louis University Hospital, St. Louis, USA

**Keywords:** gastric tumor, rare gastric tumor, chronic abdominal pain, upper endoscopy, gastric glomus tumors, glomus, gastroenterology and endoscopy, gastroenterology

## Abstract

Gastric glomus tumors (GGTs) are benign tumors that typically occur in the submucosa of the gastric wall. Glomus tumors typically occur in the subungual region of the finger and rarely manifest in the stomach. Diagnosis is challenging as these tumors lack specific clinical features, radiographic findings, and endoscopic findings. In prior cases, endoscopic ultrasound with fine-needle aspiration has been utilized to make a pre-operative diagnosis. In our case, pathology from general endoscopy was consistent with a GGT. Thus, our patient was accurately diagnosed by esophagogastroduodenoscopy (EGD) with conventional biopsy.

## Introduction

Glomus tumors result from cells of the glomus body and are typically benign. When glomus tumors occur in the stomach (i.e., gastric glomus tumors), they typically occur in the submucosa of the gastric wall. Most patients are definitively diagnosed post-operatively or have endoscopic ultrasound as these tumors lack specific clinical, radiographic, and endoscopic features. Herein, we present a case of a 44-year-old female with lower abdominal pain who was diagnosed with a mesenchymal neoplasm favoring gastric glomus tumor on biopsy specimen following esophagogastroduodenoscopy (EGD) and it was later confirmed as glomus tumor on subtotal gastrectomy specimen following laparoscopic partial gastrectomy specimen.

## Case presentation

A 44-year-old female with a past medical history of multiple sclerosis, chronic constipation, and depression presents to gastroenterology clinic for the evaluation of abdominal pain. She developed abdominal pain of the bilateral lower quadrants over the course of three months associated with intermittent nausea. She had no history of gastrointestinal bleeding. Her past surgical history is significant for a laparoscopic cholecystectomy. Her medications include fingolimod, escitalopram, and famotidine. Family history did not include a history of gastrointestinal malignancy. Physical examination showed a soft and non-tender abdomen with no other significant findings. She was started on fiber and laxatives to alleviate any component of constipation-related pain. However, in light of no improvement with these measures, the decision was made to proceed with EGD.

**Figure 1:**
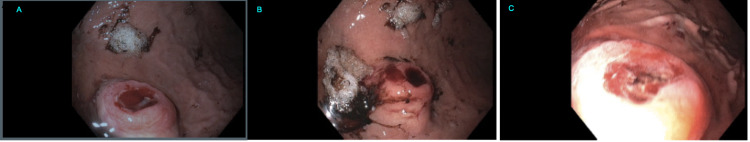
Upper endoscopy A 1.2- to 1.5-cm submucosal rounded mass with an ulcer on top with ongoing oozing and with stigmata of recent bleeding and altered hematin in much of the stomach was found in the gastric body on the greater curvature of the stomach. Biopsies were taken with a cold forceps for histology. Coagulation for hemostasis using bipolar probe was performed.

Her EGD revealed a normal esophagus and a 1.5-centimeter umbilicated gastric tumor located in the greater curvature of the stomach with a shallow ulcer on top of it that was oozing blood. The bleeding was treated with bipolar cautery, and hemostasis was achieved (Figures 1 a-1c). Grossly, this lesion was initially thought to represent a gastrointestinal stromal tumor (GIST), and biopsies were taken. A follow-up computed tomography (CT) scan showed a 2.2 x 1.8 x 2.0 cm submucosal, mixed density, well-circumscribed mass along the greater curvature of the stomach. There was no evidence of metastatic disease on imaging.

**Figure 2:**
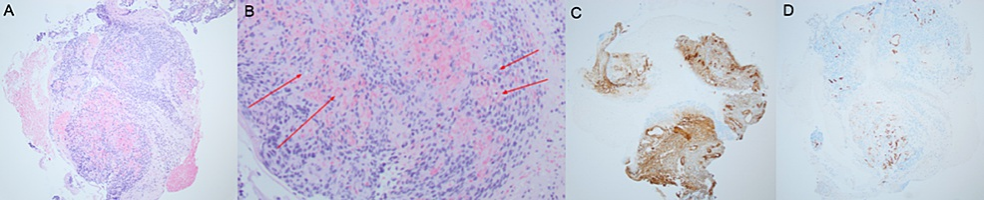
Pathology from upper endoscopy (A) A fragment of cellular neoplasm with somewhat epithelioid but syncytial appearance can be seen. (B) Red arrows that highlight prominent vasculature with hemorrhage. (C) Collagen IV immunostaining shows net-like labeling encircling each of the cells. (D) CD34 highlights numerous small vessels.

Histological examination of biopsy specimen showed multiple fragments of cellular neoplasm with somewhat epithelioid but syncytial appearance (Figure 2a) and prominent vasculature with hemorrhage (Figure 2b). Immunohistochemistry showed positive collagen IV (Figure 2c), smooth muscle actin, and vimentin along with focal desmin staining. CD117 and DOG-1 were both negative in tumor cells, strongly arguing against GIST. CD34 highlighted numerous small vessels (Figure 2d). Synaptophysin was weakly positive in a paranuclear pattern, but chromogranin, pancytokeratin, and CAM5.2 were all negative, arguing against neuroendocrine tumor. S100 was negative, which ruled out melanoma. H&E along with a pattern of positive and negative immunostatins suggested a diagnosis of mesenchymal neoplasm favoring glomus tumor.

**Figure 3:**
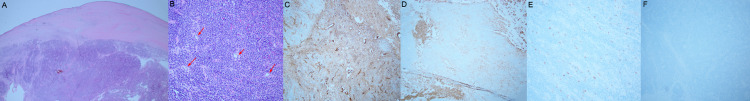
Surgical pathology (A) Low magnification shows highly cellular and fairly well-circumscribed neoplasm. (B) High magnification shows sheets of small, uniform cells with frequent cytoplasmic clearing and round central nuclei. A network of thin-walled vessels was seen throughout the tumor, consistent with glomangiopericytomatous differentiation (red arrows). (C) Collagen IV immunostaining shows net-like labeling in a pericellular distribution. (D) Calponin immunostain shows patchy and moderate reactivity. (E) CD117: very few cells are positive. (F) Negative immunoreactivity of DOG-1 argues against gastrointestinal stromal tumor.

She was referred to the surgical oncology team, and a month later, she underwent a partial laparoscopic gastrectomy with complete resection of the tumor. Histological examination of resection specimen showed a fairly well-circumscribed neoplasm within the muscularis propria with focal extension to the submucosa and mucosa (Figure 3a). The tumor comprised sheets of small, uniform cells with frequent cytoplasmic clearing, round central nuclei, with moderate, degenerative-type nuclear atypia as well as intranuclear clearing and rare intranuclear cytoplasmic inclusions (Figure 3b). Immunostains showed collagen IV in a pericellular distribution (Figure 3c). Calponin was positive, though it was patchy with moderate reactivity (Figure 3d). Desmin showed rare and patchy reactivity. Synaptophysin showed dot-like paranuclear reactivity. Very rare cells were positive for CD117 (Figure 3e). Cells were negative for chromogranin, CAM 5.2, DOG-1, and S-100, which ruled out gastrointestinal tumor, neuroendocrine tumor, and melanoma (Figure 3f).

One week after surgery, she was seen in the surgical clinic. She was still experiencing her chronic symptoms of constipation.

## Discussion

Glomus tumors are benign neoplasms that are derived from the glomus body. The glomus body is thought to participate in the thermoregulation process by facilitating the arteriovenous shunting of blood [1]. These soft tissue tumors typically develop in the upper extremities and, more specifically, have a predilection for the subungual region of the finger [2]. The tumors are composed of modified smooth muscle cells and predominantly affect women in the fifth and sixth decades of life [3]. Glomus tumors can also develop in the gastrointestinal tract, with the submucosal region of the stomach representing the most common gastrointestinal location [4]. Intramural gastric tumors, including glomus tumor, are typically mesenchymal in origin, and, overall, gastric glomus tumors represent less than 1% of all soft tissue gastric tumors, with GISTs being the most common [4].

As a group, submucosal gastric tumors can consist of both benign and malignant etiologies [5]. Gastric glomus tumors are typically benign but rarely they are considered to be malignant when meeting certain criteria. Proposed criteria for malignant glomus tumor include deep-seated location with size > 2.0 cm, or atypical mitotic figures, or moderate-to-high nuclear grade [6]. The prognostic heterogeneity of submucosal gastric tumors illustrates the importance of accurate diagnosis for these patients as different types of tumors require different modalities of management.

Clinical manifestations of gastric glomus tumors have a wide range of presentation. While some are asymptomatic and are incidentally discovered on endoscopy, others have non-specific symptoms of epigastric pain, nausea, vomiting, and gastrointestinal bleeding. A review of 56 patients showed the most common clinical manifestations were epigastric pain (61%) and blood in the stool (25%) and that 14% of patients were asymptomatic [7]. Lower abdominal symptoms are rare. Pre-operative diagnosis of gastric glomus tumors is challenging due to the lack of specific clinical, imaging, and endoscopic features. For instance, glomus tumors, GISTs, and neuroendocrine tumors all exhibit similar findings on CT scan [8]. Moreover, endoscopic biopsies often fail to provide a definitive histological diagnosis due to the inability to access sufficient tissue as deeper mucosal lesions cannot be adequately sampled [9].

While diagnosis by conventional endoscopic biopsies is possible, it is difficult. This is due in part to the intramural location of these lesions impeding sufficient specimen collection [8]. Most are submucosal without ulceration or umbilication. Therefore, endoscopic ultrasonography-guided fine needle aspiration (EUS-FNA) has been used to provide histological diagnosis in other cases [8,10]. Furthermore, a retrospective review in a population of patients with submucosal gastric tumors found that adequate specimens were obtained in 83% of cases in which patients underwent EUS-FNA and 48.9% of cases had a definitive final diagnosis [11]. Thus, EUS-FNA can play an important role in pre-operative diagnosis for these patients.

However, our patient’s endoscopy with conventional biopsy was suggestive of gastric glomus tumor, thus, EUS was not necessary in our case. Furthermore, CT scan demonstrated that the mass was contained to the stomach without any evidence of metastatic disease. Cases of gastric glomus tumor reported are managed by laparoscopic gastrectomy, which is the surgical modality chosen for our patient [12]. Findings on biopsy suggestive of gastric glomus tumor in combination with findings on CT scan of localized disease allowed for the above surgical planning for this patient. Complete resection, which was achieved in our patient, is important due to potential for malignant transformation of these tumors [13].

## Conclusions

Our case highlights two important points. First, our patient was accurately diagnosed by EGD with conventional biopsy. This was likely due in part to the growth pattern of this patient’s tumor into the gastric cavity. For this reason, EUS with FNA was deemed unnecessary and the patient was spared from another procedure. Second, the ulcerated and umbilicated appearance of tumor also emphasizes the variety of ways glomus tumor may manifest on endoscopy.

In conclusion, we presented a case of a 44-year-old woman with abdominal pain who was diagnosed with gastric glomus tumor by endoscopic biopsies and underwent laparoscopic resection. Maintaining a high index of suspicion for such tumors during endoscopy will help in achieving adequate sampling. Pre-operative diagnosis is difficult for gastric glomus tumors but is important in that it allows for more conservative workup and surgical planning.
